# The immediate effect of multiple mechanical impulses on electromyography and pressure pain threshold of lumbar latent trigger points: an experimental study

**DOI:** 10.1186/s12998-016-0101-3

**Published:** 2016-07-04

**Authors:** Bert Ameloot, Jeff Bagust

**Affiliations:** Graaf van Landaststraat 17, 9700 Oudenaarde, Belgium; Anglo-European College of Chiropractic, 13-15 Parkwood Road, Bournemouth, BH5 2DF Dorset UK

**Keywords:** Myofascial pain syndrome, Trigger point, Low back, Latent trigger point, Tender point, Surface EMG, Electromyography, Pressure pain threshold, Mechanical impulses

## Abstract

**Background:**

Myofascial pain is a common syndrome, which has not been studied extensively in the low back. Despite a variety of manual and instrument assisted interventions available, little work has targeted the possible effects of fast mechanical impulses on myofascial trigger points (MTrPs) on its sensitivity and electrical activity. The purpose of this experimental study was to quantify the immediate effect of one session of mechanical impulses to lumbar latent MTrPs and to normal muscle tissue with pressure pain threshold (PPT) and surface electromyography (sEMG) as outcome measures.

**Methods:**

During the autumn of 2009, in 41 asymptomatic subjects between 17-40 years of age the lumbar musculature was searched for a latent MTrP by a trained clinician. Using 3 disposable pre-gelled electrodes bilaterally, sEMG was recorded continuously from muscle containing either latent or no MTrP. Both the trigger point group and control group received the intervention and were blinded to group allocation. The immediate effects of mechanical impulses were assessed by sEMG and PPT before and after intervention using Wilcoxon matched-pairs signed-ranks test, Mann–Whitney *U* test and paired *t*-tests.

**Results:**

The PPT increased significantly across both groups (*p* < 0.01) after intervention. The proportionate increase (14.6 %) was comparable in both MTrP and control groups. The electrical activity on the MTrP side was not significantly higher in the MTrP group compared to the contralateral side. The decrease of resting electrical activity after intervention was significant in the MTrP group on the side of the latent MTrP (*P* = 0.001) as well as the contralateral side (*p*=0.022), and not significant in the control group on either side (*p*=0.33 and *p*=0.93).

**Conclusion:**

In this study, the immediate effect of one session of mechanical impulses was associated with a significant increase in PPT for both groups and a significant decrease in the resting electrical activity of the lumbar muscles only in the MTrP group. It is unknown if these effects have clinical significance.

## Background

Myofascial pain is a clinical problem that has generated interest, debate and confusion for decades [1–3]. According to studies anywhere between 33 and 97 % of patients with musculoskeletal pain visiting physicians and manual therapists are diagnosed with myofascial pain syndrome (MPS) [4, 5]. MPS is commonly misdiagnosed and overlooked by clinicians who are unfamiliar with this [6]. It is a complex form of neuromuscular dysfunction associated with functional deficits and broader symptomatology. It consists of soft tissue and sensory abnormalities involving both the peripheral and central nervous systems. Additionally, recent data suggest that neurogenic inflammation, wide dynamic range neurons and limbic system structures likely play pivotal roles in muscle sensitisation, pain chronification, somato-visceral interactions and the objective physical findings of allodynia, hyperalgesia and referred pain patterns [7].

MPS can be associated with, but may not be caused by, an active myofascial trigger point (MTrP) [1]. A MTrP is defined as a discrete, hyperirritable nodule in a taut band of skeletal muscle that is palpable and tender during physical examination [8, 9]. Clinically MTrPs can be classified as active or latent. Latent MTrPs may develop further into active MTrPs depending on the ongoing noxious mechanical or chemical stimulation [10–13]. An active MTrP is associated with spontaneous pain in the immediate surrounding tissue and/or to distant sites in specific referred pain patterns. A latent MTrP is not associated with spontaneous pain, however digital pressure on the nodule elicits local pain at the site and can sometimes refer pain [13]. This referral may be a result of sensitisation from long term nociceptive subthreshold signals, through opening of previously ineffective synapses [14]. Latent MTrPs show similar physical characteristics to active MTrPs, hence can also be associated with muscle stiffness, dysfunction, restricted joint range of motion, as well as autonomic dysfunction though for latent MTrPs all to a lesser degree [1, 8, 15].

The same implies for the prevalence of endplate noise or spontaneous electrical activity (SEA) being higher in active MTrPs than in the latent ones and absent in normal muscle tissue [16]. This SEA has been correlated with muscle tension and the formation of the taut band [17], pain intensity and pressure pain threshold [16]. Recent evidence suggests an important role of SEA and impaired motor control strategy at MTrPs in the induction of muscle pain and central sensitisation [18]. Besides surface electromyography (sEMG) studies on the effect of latent MTrPs on the host muscles [19], studies have shown increased electrical activity during movement of their synergists [20], decreased reciprocal inhibition of the antagonist [21] and significantly impaired muscle activation patterns during loaded and unloaded movement [22, 23]. Latent MTrP may have such an influence on muscles that could restrict athletes from performing at full capacity [22]. These characteristics and implications of latent MTrPs mentioned above make not only active but also latent MTrPs a significant concern [17].

The diagnostic accuracy of MPS had been questioned due to a paucity of studies substantiating the objectivity of physical findings. But a number of studies have now validated the physical examination findings of MTrP. These include magnetic resonance elastography [24, 25] and ultrasonoelastography [26–28]. These imaging procedures are research tools and are not clinically useful at this time. Although there is some interest in MTrP ultrasound guided needling or injection, MTrP identification by manual palpation is rapid and has proven to be reliable between trained examiners [29–32]. The criteria for diagnosis and their relative importance have evolved over time. For example, MTrP perturbation does not always produce a local twitch response or a predictable pain referral pattern, which were criteria in the past [1].

Specific to the lower back, the paraspinal muscles are commonly involved in non-specific LBP [4]. Active MTrP in muscles like the erector spinae or quadratus lumborum can be associated with pain felt as a band in the low back with occasional radiation to the buttock or into the testicles as well as difficulty with straightening up [3]. Despite the prevalence of both low back pain and MPS, MTrPs have not been studied extensively in the low back.

Current approaches for management of MPS have been reviewed elsewhere [1, 33] and include pharmacological (anti-inflammatory, analgesic, narcotic medications, topical creams, injections) and nonpharmacological interventions (manual therapies, laser therapy, dry needling). Although a number of recent reviews and meta-analyses have focused on needling, the effectiveness of manual therapy should not be overlooked, and may possibly be just as effective as needling [34]. Although many of the manual treatment methods stay the same or are only slightly modified (all include some form of mechanical pressure), it is the underlying theory as to why they are effective that continues to evolve with further study [1]. Modalities and manual treatments are often clinically effective for deactivating active MTrPs and desensitizing sensitized spinal segments [34]. They are and should be used as a first line of treatment before more invasive therapies are attempted [1]. Instrument-assisted manipulation is used as a treatment method by chiropractors to decrease stiffness of the spinal joints and muscles [35]. However, to date no studies have been done on the effects of multiple mechanical impulsive thrusts on MTrP.

## Methods

This experimental study aimed to quantify the immediate effect of one session of mechanical impulses on latent MTrPs and normal muscle tissue using pressure pain threshold (PPT) and sEMG as outcome measures. A second aim was to investigate the effect of the presence of a latent MTrPs on the electrical activity of the muscle involved compared to muscle tissue without latent MTrPs.

### Participants

Data were collected in the research laboratory at the Anglo-European College of Chiropractic (AECC) over 3 weeks during the autumn of 2009. The AECC undergraduate research panel approved this study on the 10^th^ of February 2009.

Subjects for the study were recruited from the student body of the AECC. Subjects were admitted into the study if they met the following criteria:

- Asymptomatic male or female between the age of 17–58 years old with or without a latent MTrP in the extensor group of the low back (longissimus pars lumborum, iliocostalis lumborum or quadratus lumborum). This study defined a latent MTrP as a “*discrete, hyperirritable nodule in a taut band of skeletal muscle that is palpable and tender with or without referred pain and/or local jump sign during physical examination AND that had a gender specific PPT of less than that expected in normal muscle tissue”* [22, 36–38].

Subjects were excluded from the study if any of the following was present:

- Present active MTrP
- Present low back pain or in the previous 3 months
- Known specific lumbar pathology (tumour, fracture, severe sprains, osteoporosis)
- Unable to lie prone
- Thinning skin conditions
- Evidence of nerve root or spinal cord compression
- Recent spinal surgery
- Long-term use of corticosteroid medication
- Serious neuromuscular disorders

Each subject was given a Study Information Sheet to read and was asked to sign an Informed Consent Form prior to participation in the study. The participants were blinded to the group allocation, based on the classification of having a latent MTrP or no MTrP. The examiner was partly blinded to group allocation since the criterion of gender specific muscle sensitivity was added after the experiment.

### Procedure

Once admitted to the study the subject laid prone on the treatment bench in a warm and relaxed state with the upper body disrobed. A towel was placed under the abdomen to lengthen the lumbar musculature. This creates a perceptible increase in its resistance to movement. In this position, the normal muscle fibres remain loose, but the fibres of any taut bands or nodules are placed under additional tension, thereby rendering them more easily recognizable [15]. The lumbar extensor muscles between the iliac crest and the 12^th^ rib were examined bilaterally for the presence of a latent MTrP by using cross-fibre palpation to detect any taut bands. Upon identification of a taut band, the examiner used “flat palpation” along the band looking for a focus of contraction (tender nodule) [15]. When the point was identified, the subject was asked to give feedback about the sensitivity and pain referral on digital compression. In the event of an affirmative response, the point was marked with a dermographic pencil. If multiple latent MTrP were found, the most prominent and painful MTrP was identified and selected. The identification of a latent MTrP as discussed in this study has been found to be reliable if performed on the same day by the same examiner kappa = 0.71–1.0 and intra-class correlation coefficient for PPTs = 0.92 (using test/retest protocol with 30 min between examinations) [22]. When the diagnosis is based on the combination of manual identification and gender specific sensitivity the internal validity increases [37]. If no latent MTrP was found, the exact same procedure was applied to the most tender side of the lumbar muscle 5 cm lateral to the spinous process level of L3.

#### Pressure algometry

Pressure Pain Threshold (PPT) is defined as the minimal amount of pressure where a sense of pressure first changes to pain [36]. A mechanical pressure algometer (Model PTH-AF 2, Activator Methods Inc.®, Phoenix, Arizona, USA) was used in this study. This device consists of a round, rubber-tipped plunger of 1 cm^2^ area mounted to a calibrated spring. The gauge displays values in kilogram per cm^2^ ranging from 0–10 kg with increments of 0.1 kg. The gauge held the maximum applied pressure until reset. Algometry is an objective method of quantifying soft tissue tenderness [39, 40] and has been shown to be a useful tool in the assessment of MTrP [41]. PPT measures have been found to be highly reliable and responsive to change [42–44] especially when taken by the same rater (ICC: 0.94 to 0.97) [44]. For the purposes of this study, only one measurement was taken as opposed to three repeated measures since latent MTrP may be deactivated by sustained pressure [45].

#### Surface electromyographic activity

The examination table was positioned on a screened floor (rubber floor mat with a copper mesh underneath) to reduce electrostatic interference on the sEMG recordings. The main purpose of sEMG is to monitor myoelectric manifestations of muscle fatigue, electrical and mechanical responses of single motor units as well as muscle hyperactivity [46]. In this study the electrical activity of the lumbar extensor group was recorded bilaterally at the level of L3 or across the latent TrP [47]. Following standardized skin preparation (having shaved, abraded and cleaned with alcohol), two adhesive Ag-AgCl disposable electrode pads (diameter of circular conductive area 10 mm, Kendal/Tyco Arbo H124SG, Covidien, US) placed respectively around 1 cm above and below the latent MTrP, with an interelectrode distance of 3 cm [23], being connected to the first channel [47]. The reference electrode was placed 5 cm laterally from the marked area. The positions and connections of the electrodes were copied on the contralateral side in both groups. The second channel recorded the electrical activity from the contralateral side. The six sEMG leads were firmly attached to the subject and connected to an amplifier (EMG 100C, BIOPAC systems, Inc., US) (band-pass filter: 10–500 Hz, gain: 1000, sampling rate: 1000 Hz) using a 2-channel analogue recording throughout the procedure. The BIOPAC MP150 system (42 Aero Camino, Goleta, California, 93117) was connected to a computer and the data were recorded and analysed using commercially available EMG measuring and evaluation software (AcqKnowledge 3.9, BIOPAC systems, Inc., US).

### Intervention

**Fig. 1:**
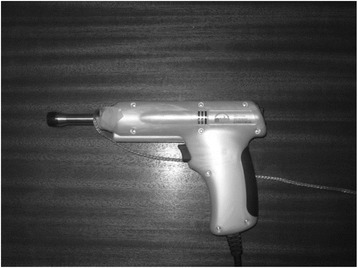
Impulse adjusting instrument set on the second force setting (200 N). The stylus base was connected by a ground wire to the screened floor to decrease interference on sEMG

Spinal manipulative instrument assistive techniques that produce very short duration (<5 ms) thrusts are termed impulsive [48]. The Impulse Adjusting Instrument® (Neuromechanical Innovations, Chandler, Arizona, USA) is a handheld, solenoid-driven electromechanical medical device (110–240 V, 50–60 Hz) that can generate extremely fast (short duration) (2 ms peak), controlled and reproducible percussion forces ranging from 100 N-400 N [49]. The intended uses of the instrument (Fig. 1) are for chiropractic adjustment, manipulation, and/or mobilization of musculoskeletal structures. The Impulse device is FDA cleared in the United States and holds a CE mark as a class II medical device in the European Union under the 93/42/EEC Medical Devices Directive. In this research experiment the second or mid impulse setting was used. On activation the instrument will thrust 1 impulse followed by 12 multiple-impulse thrust at a rate of 6.7 Hz with a force of 200 N [48]. The delivery of the thrust application requires a preload of 20 N to adequately compress the skin against the soft tissues. The stylus has a round rubber disk of 1 cm^2^ area. Taylor et al. reviewed 16 studies and concluded the use of mechanical adjusting instruments to be safe [50]. Both the participants in the MTrP group as well as the control group received the intervention as if the condition was present.

Following electrodes and leads attachment as discussed above, the subjects were asked to keep as still as possible and the rest of the procedure continued once the baseline stability of the sEMG signals appeared satisfactory. Each subject was represented by a number and gender to tag the corresponding sEMG charts. The PPT was taken with the pressure algometer on the point marked by a dermographic pencil with a rate increase in pressure of approximately 1 kg/cm^2^/s [51]. The sEMG activity was recorded for approximately 2 min. Mechanical impulses were first administered to the hand to familiarise the subject’s expectation and reduce the possibility of startle reflexes prior to the application on the lumbar muscle tissue. This was followed by the intervention that consisted of placing the instrument perpendicular to the treatment area with preload and administrating 13 impulsive thrusts as per the device indications for use. In the absence of a nodule, impulses were administered to the spot marked on the most tender side 5 cm from the midline at L3. The sEMG activity was continuously recorded for another 3 min after which the subject was asked to raise the torso off the table as a reference contraction. The post treatment PPT was recorded before the electrodes were detached.

### Data and statistical analysis

Data were analysed using SPSS 17.0 (SPSS Inc. Chicago, US) and Microsoft Office Excel (Microsoft, Washington, US). Significance level was set at *p* < 0.05 for all analyses. The lowest PPTs for male and female normal lumbar muscle were calculated [30, 36]. If a tender nodule within a palpable taut band was found with a pre-treatment PPT below 5.7 kg (male) or 4.2 kg (female), the subject assigned to the MTrP group. If the PPT was higher than these thresholds or if there was no palpable taut band with a tender nodule the subject was allocated to the control group.

An unpaired *t*-test with Welch correction was used to compare the age and the pre-treatment PPT between males and females. The mean difference between PPT pre- and post-treatment in the MTrP and control group were compared using a paired *t*-test, since the data were normally distributed. The difference in the change in PPT values between the MTrP group and the control group was calculated using the unpaired *t*-test with Welch correction.

The 2 and 3 min of raw EMG recordings pre- and post-intervention were screened by visually scanning the signal for artifacts and stability. Then a period of 20 s was selected bilaterally pre- and post-intervention that was representative of rest (stable low electrical activity, without interferences). A high-pass filter at 30 Hz could have been applied to the signal to reduce the artefact of electrocardiographic interference, however in this study this may remove useful information. Therefore, heart rate variability was assessed by determining the heart rate pre- and post-intervention on the raw 20-s interval sEMG traces representing a resting state (paired *t*-test). The bilateral raw EMG signal was amplified, filtered (high pass 10 Hz; low pass = 500 Hz) and subjected to a root mean square (RMS) protocol (window 100 ms; Acqknowledge 3.9, Biopac). The mean amplitude value or average of the data samples between the endpoints of the selected area (20 s periods) of the RMS waveforms recorded before and after intervention were computed bilaterally. The sEMG data of both the latent MTrP group and control group were analysed for the differences pre- and post-intervention on each side (non-parametric Wilcoxon matched-pairs rank test) and between the MTrP side and the contralateral side in the same subject in one group (non-parametric Wilcoxon matched-pairs signed-ranks test). The reference contraction data were not used to normalise the EMG data since there were too many missing values in the control group, increasing variability and decreasing generalisability if left out in the analysis.

## Results

**Fig. 2:**
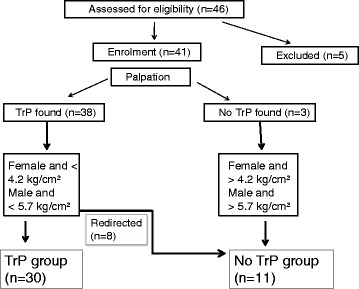
Flowchart of the study presenting the final group allocation

Five subjects were excluded due to current low back pain, leaving 41 participants that met all selection criteria and completed the study (Fig. 2). The sample consisted of 25 males (61 %) with mean (SD) for age 24 (5.4) and pre-intervention PPT 4.5 kg/cm^2^ (1.5) and 16 females (39 %) with mean (SD) for age 22 (3.0) and pre-intervention PPT 3.7 kg/cm^2^ (1.2). The PPT between males and females was considered not significant in this sample (*P* = 0.087; 95 % CI (-0.1 to 1.6). In 38 subjects (92 %) a latent MTrP was found based on palpation criteria alone. Eight subjects had a PPT that was higher than expected in normal muscle tissue and were during analysis redirected to the control group (Fig. 2). Based on both palpation and gender specific PPT criteria a latent MTrP was present in 73.2 % of subjects (the MTrP group).

**Fig. 3:**
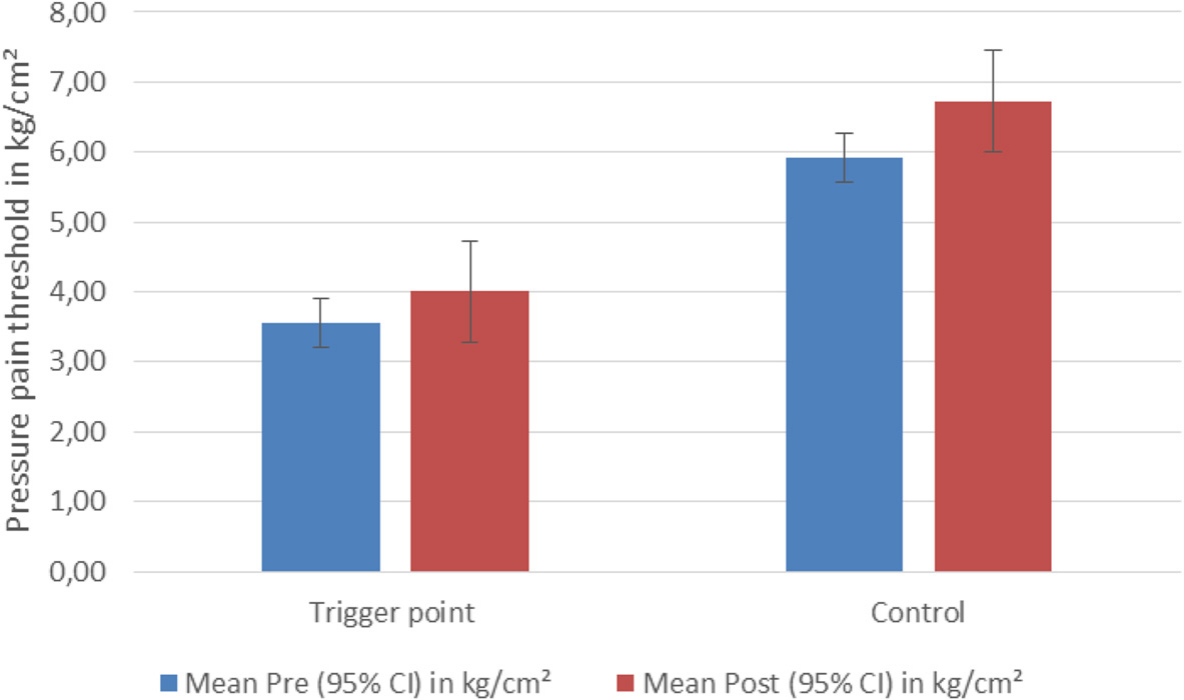
Comparison of pressure pain threshold pre- and post-intervention in the trigger point group (*n* = 30) versus the control group (*n* = 11)

The mean post-intervention increase in PPT was significant for both the MTrP group (0.45 kg/cm^2^; *P* = 0.01; CI 0.11-0.8 kg/cm^2^) and the control group (0.82 kg/cm^2^; *P* = 0.03; CI 0.09–1.54). The increase in PPT values between the MTrP group and the control group was found to be similar (*P* = 0.33), using the unpaired *t*-test with Welch correction (Fig. 3 and Table 1).

**Table 1:** Comparison of the mean and significance of the differences in pressure pain threshold pre-and post-intervention in the trigger point group (*n* = 30) versus the control group (*n* = 11) versus all subjects (*n* = 41).

	Trigger point	Control	All subjects
	*n* = 30	*n* = 11	*n* = 41
Mean Pre (95 % CI) in kg/cm^2^	3.55 (3.20–3.90)	5.91 (5.18–6.64)	4.18 (3.73–4.64)
Mean Post (95 % CI) in kg/cm^2^	4.00 (3.56–4.44)	6.73 (5.77–7.68)	4.73 (4.18–5.29)
Difference (95 % CI) in kg/cm^2^	0.45 (0.11–0.80)	0.82 (0.09–1.54)	0.55 (0.25–0.85)
Change in proportion	14.60 % (3.09–26.12)	14.30 % (2.11–26.49)	14.52 % (5.76–23.29)
*P* value	0.011	0.030	0.001

CI (Confidence Interval) Change in proportion (mean of (individual difference divided by the individual pre-intervention value))

The mean pre-intervention resting electrical activity of the MTrP group (*n* = 30) were not significantly different between the MTrP side and the contralateral side (3.27 μV; SD 3.64 and 3.1 μV; SD 3.36 respectively; *P* = 0.3). Similarly, in the control group (*n* = 11) this mean pre- intervention resting electrical activity on the intervention side (1.67 μV; SD 1.02) was not significantly different from the contralateral side (1.4 μV; SD 1.13; *P* = 0.33).

According to the rank test there was a significant (*P* = 0.001) change in the resting electrical activity post-intervention on the latent MTrP side in the MTrP group (in 23 subjects the EMG activity decreased post-intervention, 7 increased) the mean decreased from 3.27 μV (SD ± 3.64) to 2.69 μV (SD ± 2.75) (Table 2). In the control group this was not significantly decreased (*P* = 0.33) from 1.67 μV (SD 1.02) to 1.40 μV (SD 1.13). The contralateral side in the MTrP group did also decrease significantly (*P* = 0.022), from 3.1 μV (SD 3.36) to 2.77 μV (SD 2.95), the amplitude decreased in 19 subjects and 9 subjects increased. The changes were non-significant (*P* = 0.93) in the control group 1.77 μV (SD 1.07) to 1.76 μV (SD 1.5), 7 subjects had decreased electrical activity post-intervention and 4 increased.

**Table 2:** Presentation of the mean resting sEMG pre- and post-intervention in the trigger point group (*n* = 30) and the control group (*n* = 11).

sEMG in μV	Trigger point group	Control group
Baseline mean intervention side (±SD)	3.27 (SD 3.64)	1.67 (SD 1.02)
Baseline mean contralateral side (±SD)	3.1 (SD 3.36)	1.77 (SD 1.07)
Post-intervention mean intervention side (±SD)	2,69 (SD 2.75)	1.40 (SD 1.13)
Post-intervention mean contralateral side (±SD)	2.77 (SD 2.95)	1.76 (SD 1.5)
Pre-post change significance intervention side	*P* = 0.001	*P* = 0.33
Pre-post change significance contralateral side	*P* = 0.022	*P* = 0.93

Statistical significance was tested via the non-parametric Wilcoxon matched-pairs rank test. SD (Standard deviation)

The mean heart rate pre-intervention was 65 beats per minute (SD 10) and post-intervention 64 beats per minute (SD 9). The difference of the heart rate between pre-and post-intervention was not significant (*P* = 0.09) and 95 % CI (−0.2–2.4).

## Discussion

To our knowledge, this is the first study to evaluate the effects of latent MTrP in the low back using sEMG. It is also the first study that looks at the effect of multiple mechanical impulsive thrusts on MTrPs in the low back. This experiment and its results should be viewed as a preliminary study.

The presence of latent TrPs is common in the general population [3, 10, 15, 33, 51, 52]. We found at least one latent MTrP in the low back in 73 % of asymptomatic subjects, which is high compared to other studies [53–55]. For example, the lumbo-gluteal muscles in 100 asymptomatic control subjects were examined and 45 % of the subjects were found to have latent MTrPs in the quadratus lumborum muscle [53]. The point prevalence, in a more recent study of latent MTrPs in the quadratus lumborum and iliocostalis lumborum muscles in asymptomatic controls was between 5 % and 10 %, while latent MTrPs were found in the same muscles mentioned on each side in 15 to 20 % of patients with chronic nonspecific low back pain (*n* = 42) [54]. When looking at other regions, 154 asymptomatic adults were examined for the presence of latent TrPs in the scapular positioning muscles and approximately 90 % of this population had at least one trigger point in these muscles [52]. Another study found that latent TrPs produced focal tenderness in shoulder girdle muscles in 54 % of female participants and in 45 % of male participants [56]. Of these participants *n* = 200, 25 % exhibited referred pain on digital compression. Referred pain is however more commonly encountered when inserting a needle in a latent MTrP than firm compression and is not common in normal muscle tissue [21]. In our study 5 subjects (17 %) exhibited referred pain on digital compression, while none in the control group.

Several reasons could explain the difference in the point-prevalence of MTrPs found in this study compared to others mentioned above. One of them is that reproducibility, in this context, is not strictly speaking confined to the skill of manual palpation. In fact, only the identification of the taut band is purely a touch skill. The other MTrP diagnostic criteria are either observations (local twitch response and jump sign), established through patient feedback (pain referral and patient pain recognition), or are a hybrid (local tenderness is felt for but confirmed by patient feedback) [57]. Secondly, MTrP criteria do not appear universally present in every muscle group [1]. Thirdly this population might be different to the ones in the other studies as students may have had a period of increased lumbar muscular loading.

### Post-intervention increase in pressure pain threshold

In our study male subjects (*n* = 25) had a statistically non-significant higher PPT (+0.8 kg) than the female participants (*n* = 16). This was comparable to the study of Chesterton et al. [38] who found a significant difference of 1.2 kg between the genders. Hence to determine group allocation we used a sensitivity threshold of the tissue that was gender specific. The mean increases of PPT after intervention were comparable and significant for both the MTrP 0.45 kg/cm^2^ and control group 0.82 kg/cm^2^. The proportionate increase of PPT was the same in both groups (14 %). An explanation for this phenomenon could be the desensitisation of the skin and myofascial tissue via rapidly adapting phasic mechanoreceptors. However, when an ischaemic block was placed at a non-MTrP region no significant change in PPT was noted at the different measurement times pre-during and post-intervention [58, 59]. Consequently, having measured 9 times the same non-MTrP region by mechanical compression (pressure algometry) the change in sensitivity of the tissue was non-significantly different [59], which might decrease the likelihood of the first suggested hypothesis. The general increase in PPT could also be explained by the ‘pain gate theory’ where afferent mechanical input from the impulsive thrusts on the skin and myofascial tissue inhibit nociceptive transmission from thin (Aẟ and C) fibers at the dorsal horn of the spinal cord via inhibitory interneurons and presynaptic inhibition [60, 61]. Various experimental research articles on deactivation of MTrPs suggest that the decrease in pain is due to this gating mechanism [59–63]. Another explanation would be the central nervous system pain-modulatory mechanism called diffuse noxious inhibitory control [64, 65]. This descending anti-nociceptive pathway (also known as counter-irritation) is evoked by nociceptive stimuli (i.e. heat, high pressure, electrical stimulation) that ascends from the spinal cord to the brain. In turn, the brain inhibits pain transmission monoaminergically [64, 65], which leads to reduced pain perception not only locally but also at distant sites [65]. Depending on the sensitization of the muscle tissue, the mechanical thrusts can be interpreted by the nervous system as mechanical and/or noxious.

Besides the neurological explanation, mechanical effects have been proposed based on the physical changes of MTrPs. The following steps are supposedly involved. A decrease in length of the affected muscle fibers along with a local muscle spasm promotes vasoconstriction. The consequent induction of a hypoxic state at the affected area of the muscle possibly creates peripheral sensitisation via insufficient adenosinetriphosphate synthesis in type I motor unit fibers, increasing acidity, free calcium accumulation and subsequent sarcomere contracture [1, 66, 67]. MTrP compression or repeated mechanical impulses may cause an increase in local blood circulation [68–70]. Therefore, the increased availability of adenosine triphosphate helps to decrease the local spasm at the myofascial active loci, achieve an energetically adequate metabolic state as well as a decrease of inflammatory mediators locally [66, 68–70]. It is not known which mechanisms play a role and to what extent. This process may also be responsible for the decrease of excess acetylcholine in the synaptic cleft and consequent decrease in SEA [67]. The mechanical impulses used in this experiment have been shown in both animal models and humans to create appreciable vertebral motions [66, 71] and electromyographic responses [72–74] thought to be responsible for underlying mechanisms of clinical benefits of spinal manipulation in low back pain patients. Other related clinical work has also found a reduction in neck pain following ten repeated impulsive thrusts with a spring-loaded device in patients with trapezius active MTrPs [70]. Lidocaine MTrP injections combined with MTrP compression (30 s or 60 s) showed better effects on treatment of MTrP in the upper trapezius muscle than MTrP injection therapy alone [75]. This confirms the necessary mechanical compression of the MTrP area. Besides an increase in blood flow another purpose of mechanical compression of a MTrP is to lengthen the shortened muscle fibres through impulses on the localised contraction [67, 69]. It is unknown whether this effect is also due to spinal mechanisms [68]. Sustained nociceptive mechanical stimulation by means of intramuscular needle movement for 8 min only produced mechanical hyperalgesia in the latent MTrP, which was not seen in the non-TrP area [13]. In our study the intervention was the application of repeated mechanical impulsive thrusts with a pressure of 200 Newton, which is a multiple of the PPT. So the length of the mechanical stimulation (needle or impulses) might play a role [13], since active MTrPs can be deactivated via short needling [1, 7, 15, 33, 34].

### Baseline surface electromyography

In our study we noticed a high variability of resting EMG activity within and between individuals, which has been described in the past when normal and tender lumbar musculature were evaluated [76, 77]. For this reason, the non-parametric Wilcoxon matched-pairs rank test is useful, because it evaluates the change in the individual subject. The electrode placement was fairly consistent with the location of the MTrP at around L3 spinal level, so variation of multifidus activity that can occur at different spinal levels would have been minimal [76].

The mean resting electrical activity at the MtrP side in the MTrP group at baseline was 3.27 μV (SD 3.64) and 1.67 μV (SD 1.02) in the control group. No statistical comparison can be made, as these are non-normalised values. Other studies have evaluated if surface and/or intramuscular EMG recordings of MTrPs are different from normal muscle tissue during rest or activity. In a study (*n* = 87) with headache patients the upper trapezius and sternocleidomastoid muscles showed higher amplitudes of intramuscular EMG activity in muscles with compared to without latent MTrPs [78]. This was confirmed more recently in latent MTrPs of the deltoid muscle during rest and movement [21]. Active MTrPs in the erector spinae muscles exhibited 2–3 times higher resting sEMG recordings compared to the control group without MTrPs [79]. The study of Ge et al. [10] however showed similar increased spontaneous electrical activity (SEA) at the MTrPs via intramuscular EMG and surface EMG measurements [80]. Even though generally the EMG of MTrPs are increased, conflicting data exists with regards to latent MTrPs when measured via sEMG during rest and loading [20]. The SEA in a latent MTrP, which is not present in non-MTrP regions [81] seems to be correlated with the degree of irritability and is therefore even higher in active MTrP [16]. It is believed that this increased motor unit excitability contributes significantly to muscle tension and the formation of a taut band associated with MTrP [13, 82] as well as the induction of local tenderness and pain upon mechanical stimulation and the local motor dysfunctions such as muscle cramps and weakness, restricted joint range of motion, impaired motor control strategy and accelerated fatigability [23, 80, 83, 84]. It is unknown whether spinal inhibitory mechanisms are related to the increased motor unit excitability at MTrPs [21].

### Decreased electrical activity of the MTrP after mechanical impulsive thrusts

The decrease of resting electrical activity in the MTrP group was not due to a change in heart activity before and after the application of the impulsive thrusts (*P* = 0.09). In the current study, the decrease of sEMG activity after intervention was significant (*P* = 0.001) on the MTrP side in the MTrP group and not in the control group on the intervention side nor the contralateral side (*P* = 0.33 and *P* = 0.93 respectively). This change might be indicative of the immediate effects of repeated mechanical impulses on a latent MTrP through mechanisms discussed above [68–70]. It is interesting to note that there was also a statistically significant decrease of electrical activity on the contralateral side (*P* = 0.022) only in the MTrP group suggesting motor endplate modulation by muscle spindle afferents and sympathetic hyperactivity influencing local or supraspinal reflex changes on muscular activity following this type of afferent input [80].

The findings in our study are not only supported by animal studies that have found immediate reductions in SEA after dry needling [63, 85], but also in human studies [86, 87]. Koppenhaver et al. (2015) measured via ultrasound the multifidus muscle thickness at rest and during contraction after dry needling the MTrP and control area. A decrease in resting thickness after dry needling would be consistent with an inhibition of excessive muscle activity (i.e. the alleviation of muscle “spasm”). They found an increased percent thickness change that appears to have been driven predominantly by an increased contracted thickness rather than a decrease in resting thickness, suggesting a facilitation of muscle contraction rather than an inhibition of resting muscle activity [86]. In another study the decrease in electrical activity after MTrP compression and ultrasound of the MTrP was related with the improvement of active cervical range of motion and decreasing the MTrPs sensitivity [87]. Others have also observed changes in EMG activity following various MTrP interventions [67, 88–90]. Hendler et al. reported a statistically significant decrease in trapezius muscle activity with MPS (*n* = 18, no control group) measured using sEMG following injection of a local anaesthetic in the MTrP, which accompanied the subjective relief reported by the patients [88]. Reductions in resting sEMG activity were recorded at the site of referred pain (masseter) after MTrP injection of a lidocaïne solution in the upper trapezius [89]. Another study (*n* = 16) did not to reach statistical significance in decreased trapezius muscle activity at the MTrP site after injection of lidocaine [90]. However, in that study the baseline resting muscle activity decreased significantly at the control site (2 cm from the MTrP) on the upper trapezius [90]. In a larger study both SEA and sensitivity were significantly reduced after MTrP compression or passive stretching, especially when combined [67]. These results were similar to the findings obtained in this experiment.

### Study limitations

There may have been interpretation and examiner bias as the examiner also analysed the data. Another issue is the possibility of cross talk from other muscles that cannot be avoided by using this type of EMG recording. The reference contraction should have been done on all subjects to be able to compare all variables between groups with normalised values. Besides this, it is still not clear whether latent MTrPs have an effect on the strength of the muscle involved [91, 92]. This muscular effort can influence sEMG in asymptomatic subjects [93]. Secondly, PPTs can remain elevated for 10 min after a static endurance test. Therefore, we did not want to influence PPTs nor sEMG because of maximally challenging the muscle by the use of a maximally voluntary contraction.

It is possible that subjects have other latent MTrP(s) on the same or on the contralateral side. This was not a problem when comparing pre- to post-intervention. The variability of the inter subject response to PPT could also be a source of error when looking at the means. However, in this regard all subjects were given the same clear instructions and the discrepancy between pain threshold and pain tolerance was explained to the subjects.

The effects of one session of mechanical impulses found in this study may or may not be clinically relevant. There is still some suspicion that the changes seen in this study may be a normal reaction to the intervention regardless of a latent MTrP or not. Therefore, further work on symptomatic individuals is necessary to assess the effect of multiple impulses on active MTrPs, while incorporating sham impulses to elucidate their clinical effectiveness in musculoskeletal conditions. Future studies should not compare painful versus normal muscle tissue, rather muscle tissue containing latent versus active MTrP versus normal muscle tissue. This will help identify the changes in the pathophysiological spectrum and decrease possible bias of muscle reflex and pain inhibition.

## Conclusion

On the basis of the results from this study one session of mechanical impulses was associated with a decrease in pain sensitivity in the low back musculature regardless of whether the muscle was normal or contained a latent trigger point. The intervention was associated with a significant decrease in the resting electrical activity solely in the trigger point group. This study should be considered exploratory in nature.

### Availability of data and materials

The dataset supporting the conclusions of this article is included within the article and its additional files.

## Abbreviations

AECC: Anglo-European College of Chiropractic
CI: confidence interval
MPS: myofascial pain syndrome
MTrP: myofascial trigger point
PPT: pressure pain threshold
RC: reference contraction
SD: standard deviation
SEA: spontaneous electrical activity
sEMG: surface electromyography

## References

[1] Shah JP, Thaker N, Heimur J, Aredo JV, Sikdar S, Gerber L (2015). Myofascial Trigger Points Then and Now: A Historical and Scientific Perspective. PM R.

[2] Quintner JL, Bove GM, Cohen ML (2015). A critical evaluation of the trigger point phenomenon. Rheumatology (Oxford).

[3] Dommerholt J, Gerwin RD (2015). A critical evaluation of Quintner et al.: missing the point. J Bodyw Mov Ther.

[4] Rickards LD (2006). The effectiveness of non-invasive treatments for active myofascial trigger point pain: A systematic review of the literature. Int J Osteopathic Med.

[5] Harden RN, Bruehl SP, Gass S, Niemiec C, Barbick B (2000). Signs and symptoms of the myofascial pain syndrome: a national survey of pain management providers. Clin J Pain.

[6] Raj PP, Paradise LA (2004). Myofascial pain syndrome and its treatment in low back pain. Semin Pain Med.

[7] Srbely J (2010). New trends in the treatment and management of myofascial pain syndrome. Curr Pain Head Rep.

[8] Simons DG, Travell JG, Simons LS (1999). Myofascial pain and dysfunction: the trigger point manual, vol. 1.

[9] Bennett R (2007). Myofascial pain syndromes and their evaluation. Best Pract Res Clin Rheum.

[10] Ge H-Y, Arendt-Nielsen L (2011). Latent myofascial trigger points. Curr Pain Head Rep.

[11] Ge H-Y, Zhang Y, Boudreau S, Yue SW, Arendt-Nielsen L (2008). Induction of muscle cramps by nociceptive stimulation of latent myofascial trigger points. Exp Brain Res.

[12] Zhang Y, Ge H, Yue S, Kimura Y, Arendt-Nielsen L (2009). Attenuated Skin Blood Flow Response to Nociceptive Stimulation of Latent Myofascial Trigger Points. Arch Phys Med Rehab.

[13] Xu YM, Ge HY, Arendt-Nielsen L (2010). Sustained nociceptive mechanical stimulation of latent myofascial trigger point induces central sensitization in healthy subjects. J Pain.

[14] Mense S (2010). How do muscle lesions such as latent and active trigger points influence central nociceptive neurons?. J Musculoskelet Pain.

[15] Celik D, Mutlu EK (2013). Clinical Implication of Latent Myofascial Trigger Point. Curr Pain Headache Rep.

[16] Kuan T-S, Hsieh Y-L, Chen S-M, Chen J-T, Yen W-C, Hong C-Z (2007). The myofascial trigger point region: correlation between the degree of irritability and the prevalence of endplate noise. Am J Phys Med Rehabil.

[17] Jafri MS (2014). Review Article Mechanisms of Myofascial Pain. Internat Schol Res Not.

[18] Ge H-Y, Fernández-de-las-Peñas C, Yue SW (2011). Myofascial trigger points: spontaneous electrical activity and its consequences for pain induction and propagation. Chin Med.

[19] Gemmell H, Bagust J (2009). Can surface electromyography differentiate muscle activity between upper trapezius muscles with active versus latent trigger points? A cross-sectional study. Clin Chiropr.

[20] Ge H-Y, Monterde S, Graven-Nielsen T, Arendt-Nielsen L (2014). Latent myofascial trigger points are associated with an increased intramuscular electromyographic activity during synergistic muscle activation. J Pain.

[21] Ibarra JM, Ge H-Y, Wang C, Martínez Vizcaíno V, Graven-Nielsen T, Arendt-Nielsen L (2011). Latent myofascial trigger points are associated with an increased antagonistic muscle activity during agonist muscle contraction. J Pain.

[22] Lucas KR. The effects of latent myofascial trigger points on muscle activation patterns during scapular plane elevation. Thesis, (PhD). School of Health Sciences Royal Melbourne Institute of Technology 2007

[23] Lucas KR, Rich PA, Polus BI (2010). Muscle activation patterns in the scapular positioning muscles during loaded scapular plane elevation: the effects of latent myofascial trigger points. Clin Biomech.

[24] Chen Q, Bensamoun S, Basford J (2007). Identification and quantification of myofascial taut bands with magnetic resonance elastography. Arch Phys Med Rehabil.

[25] Chen Q, Basford J, An KN (2008). Ability of magnetic resonance elastography to assess taut bands. Clin Biomech.

[26] Sikdar S, Shah JP, Gilliams E (2008). Assessment of myofascial trigger points (MTrPs): a new application of ultrasound imaging and vibration sonoelastography. Conf Proc IEEE Eng Med Biol Soc.

[27] Turo D, Otto P, Shah JP (2012). Ultrasonic tissue characterization of the upper trapezius muscle in patients with myofascial pain syndrome. Conf Proc IEEE Eng Med Biol Soc.

[28] Sikdar S, Shah JP, Gebreab T (2009). Novel applications of ultrasound technology to visualize and characterize myofascial trigger points and surrounding soft tissue. Arch Phys Med Rehabil.

[29] Gerwin RD, Shannon S, Hong CZ (1997). Interrater reliability in myofascial trigger point examination. Pain.

[30] Sciotti VM, Mittak VL, DiMarco L (2001). Clinical precision of myofascial trigger point location in the trapezius muscle. Pain.

[31] Bron C, Franssen J, Wensing M, Oostendorp RA (2007). Interrater reliability of palpation of myofascial trigger points in three shoulder muscles. J Man Manip Ther.

[32] Hua NK, Van der Does E (1994). The occurrence and inter-rater reliability of myofascial trigger points in the quadratus lumborum and gluteus medius: A prospective study in non-specific low back pain patients and controls in general practice. Pain.

[33] Maijlesi J, Unalan H (2010). Effect of treatment on trigger points. Curr Pain Headache Rep.

[34] Rayegani SM, Bayat M, Bahrami MH, Raeissadat SA, Kargozar E (2014). Comparison of dry needling and physiotherapy in treatment of myofascial pain syndrome. Clin Rheumatol.

[35] Fuhr AW, Menke JM (2005). Status of Activator Methods Chiropractic Technique, Theory, and Practice. J Manip Phys Ther.

[36] Fischer AA (1990). Application of pressure algometry in manual medicine. J Manual Med.

[37] Imamura M, Chen J, Matsubayashi SR, Targino RA, Alfieri FM, Bueno DK, Hsing WT (2013). Changes inpressure pain threshold in patients with chronic nonspecific low back pain. Spine (Phila Pa 1976).

[38] Chesterton LS, Barlas P, Foster NE, Baxter GD, Wright CC (2003). Gender differences in pressure pain threshold in healthy humans. Pain.

[39] Schneider M, Hyde TE, Ko G, Lawson G, Perera J, Pfefer M, Vernon H. Literature synthesis: chiropractic management of soft tissue conditions. Final draft. Chiropr Clin Compass. 2007, CCGPP.

[40] Potter L, McCarthy C, Oldham J (2006). Algometer reliability in measuring pain pressure threshold over normal spinal muscles to allow quantification of anti-nociceptive treatment effects. Internat J Osteo Med.

[41] Vanderweeën L, Oostendorp RAB, Vaes P, Duquet W (1996). Pressure algometry in manual therapy. Man Ther.

[42] Potter L, McCarthy C, Oldham J (2006). Algometer reliability in measuring pain pressure threshold over normal spinal muscles to allow quantification of anti-nociceptive treatment effects. Int J Osteopath Med.

[43] Kinser A, Sands W, Stone M (2009). Reliability and validity of a pressure algometer. J Strength Cond Res.

[44] Walton DM, Macdermid JC, Nielson W, Teasell RW, Chiasson M, Brown L (2011). Reliability, standard error, and minimum detectable change of clinical pressure pain threshold testing in people with and without acute neck pain. J Orthop Sports Phys Ther.

[45] Hou C-R, Tsai L-C, Cheng K-F, Chung K-C, Hong C-Z (2002). Immediate effects of various physical therapeutic modalities on cervical myofascial pain and trigger-point sensitivity. Arch Phys Med Rehab.

[46] Ginn KA, Halaki M (2015). Do surface electrode recordings validly represent latissimus dorsi activation patterns during shoulder tasks?. J Electromyogr Kinesiol.

[47] Cram JR, Kasman GS, Holtz J (1998). Introduction to surface electromyography.

[48] Keller TS, Colloca CJ, Fuhr AW (1999). Validation of the force and frequency characteristics of the activator adjusting instrument: effectiveness as a mechanical impedance measurement tool. J Manipulative Physiol Ther.

[49] Colloca CJ, Keller TS, Black P, Normand M, Harrison DE, Harrison DD (2005). Comparison of mechanical force of manually assisted chiropractic adjusting instruments. J Manipulative Physiol Ther.

[50] Taylor SH, Arnold ND, Biggs L, Colloca CJ, Mierau DR, Symons BP, Triano JJ (2004). A review of the literature pertaining to the efficacy, safety, educational requirements, uses and usage of mechanical adjusting devices. Part 2 of 2. J Can Chiropr Assoc.

[51] Ibàñez-García J, Alburquerque-Sendín F, Rodríguez-Blanco C, Girao D, Atienza- Meseguer A, Planella-Abella S, Fernàndez-de-las Peñas C (2009). Changes in masseter muscle trigger points following strain-counterstrain or neuro-muscular technique. J Body Movem Ther.

[52] Lucas KR, Rich PA, Polus BI (2008). How common are latent myofascial trigger points in the scapular positioning muscles. J Musculoskelet Pain.

[53] Fröhlich D, Fröhlich R (1995). Das Piriformis syndrom: eine häufige differential diagnose des lumboglutäalen schmerzes. Man Med.

[54] Iglesias‐González JJ, Muñoz‐García MT, Rodrigues‐de‐Souza DP, Alburquerque Sendín F, Fernández-de‐las‐Peñas C (2013). Myofascial trigger points, pain, disability, and sleep quality in patients with chronic nonspecific low back pain. Pain Med.

[55] Chiarotto A, Clijsen R, Fernandez-de-las-Penas C, Barbero M. The Prevalence of Myofascial Trigger Points in Spinal Disorders: a Systematic Review and Meta-Analysis. Arch of Phys Med and Rehab. Available online 17 October 2015. In Press 10.1016/j.apmr.2015.09.021 26475933

[56] Sola AE, Rodenberger ML, Gettys BB (1955). Incidence of hypersensitive areas in posterior shoulder muscles; a survey of two hundred young adults. Am J Phys Med.

[57] Myburgh C, Larsen AH, Hartvigsen J (2008). A systematic, critical review of manual palpation for identifying myofascial trigger points: evidence and clinical significance. Arch Phys Med Rehabil.

[58] Li LT, Ge H-Y, Yue SW, Arendt-Nielsen L (2009). Nociceptive and non-nociceptive hypersensitivity at latent myofascial trigger points. Clin J Pain.

[59] Wang YH, Ding XL, Zhang Y (2010). Ischemic compression block attenuates mechanical hyperalgesia evoked from latent myofascial trigger points. Exp Brain Res.

[60] Wall PW, Melzack R (1984). Textbook of Pain.

[61] Melzack R (1996). Gate control theory: on the evolution of pain concepts. Pain Forum.

[62] Meng F, Ge HY, Wang YH, Yue SW (2015). A afferent fibers are involved in the pathology of central changes in the spinal dorsal horn associated with myofascial trigger spots in rats. Exp Brain Res.

[63] Hsieh YL, Chou LW, Joe YS, Hong CZ (2011). Spinal cord mechanism involving the remote effects of dry needling on the irritability of myofascial trigger spots in rabbit skeletal muscle. Arch Phys Med Rehabil.

[64] Le Bars D, Villanueva L, Bouhassira D, Willer JC (1992). Diffuse noxious inhibitory controls (DNIC) in animals and in man. Patol Fiziol Eksp Ter.

[65] Aboodarda SJ, Spence AJ, Button DC (2015). Pain pressure threshold of a muscle tender spot increases following local and non-local rolling massage. BMC Musculoskelet Disord.

[66] Shah JP, Gilliams EA (2008). Uncovering the biochemical milieu of myofascial trigger points using in vivo microdialysis: an application of muscle pain concepts to myofascial pain syndrome. J Bodyw Mov Ther.

[67] Kostopoulos D, Nelson AJ, Ingber RS, Larkin RW (2008). Reduction of spontaneous electrical activity and pain perception of trigger points in the upper trapezius muscle through trigger point compression and passive stretching. J Musculosk Pain.

[68] Gemmell H, Allen A (2008). Relative immediate effect of ischaemic compression and activator trigger point therapy on active upper trapezius trigger points: a randomised trial. Clin Chiropr.

[69] Gemmell H, Miller P, Nordstrom H (2008). Immediate effect of ischaemic compression and trigger point pressure release on neck pain and upper trapezius trigger points: a randomised controlled trial. Clin Chiropr.

[70] Blikstad A, Gemmell H (2008). Immediate effect of activator trigger point therapy and myofascial band therapy on non-specific neck pain in patients with upper trapezius trigger points compared to sham ultrasound: a randomised controlled trial. Clin Chiropr.

[71] Keller TS, Colloca CJ, Moore RJ, Gunzburg R, Harrison DE (2006). Increased multiaxial lumbar motion responses during multiple-impulse mechanical force manually assisted spinal manipulation. Chiropr Osteopat.

[72] Colloca CJ, Keller TS, Gunzburg R (2004). Biomechanical and neurophysiological responses to spinal manipulation in patients with lumbar radiculopathy. J Manipulative Physiol Ther.

[73] Colloca CJ, Keller TS (2001). Electromyographic reflex response to mechanical force, manually-assisted spinal manipulative therapy. Spine.

[74] Colloca CJ, Keller TS, Harrison DE, Moore RJ, Gunzburg R, Harrison DD (2006). Spinal manipulation force and duration affect vertebral movement and neuromuscular responses. Clin Biomech.

[75] Kim SA, Oh KY, Choi WH, Kim IK (2013). Ischemic compression after trigger point injection affect the treatment of myofascial trigger points. Annals Rehab Med.

[76] Lee L, Coppieters MW, Hodges P (2005). Differential activation of the thoracic multifidus and longissimus thoracis during trunk movement. Spine.

[77] Fryer G, Morris T, Gibbons P, Briggs A (2006). The electromyographic activity of thoracic paraspinal muscles identified as abnormal with palpation. J Manipulative Physiol Ther.

[78] Donaldson CCS, Skubick DL, Clasby RG, Cram JR (1994). The evaluation of trigger-point activity using dynamic EMG techniques. Am J Pain Med.

[79] Wytra̦żek M, Huber J, Lisiński P (2011). Changes in muscle activity determine progression of clinical symptoms in patients with chronic spine-related muscle pain. A complex clinical and neurophysiological approach. Funct Neurol.

[80] Ge HY, Arendt-Nielsen L (2011). Latent myofascial trigger points. Curr Pain Headache Rep.

[81] Simons DG, Hong C-Z, Simons LS (1995). Prevalence of spontaneous electrical activity at trigger spots and at control sites in rabbit skeletal muscle. J Musculoskelet Pain.

[82] Gerwin R (2010). Myofascial pain syndrome: Here we are, where must we go. J Musculoskelet Pain.

[83] Ge HY, Arendt-Nielsen L, Madeleine P (2012). Accelerated muscle fatigability of latent myofascial trigger points in humans. Pa Med.

[84] Meng F, Ge HY, Wang YH, Yue SW (2015). Myelinated Afferents Are Involved in Pathology of the Spontaneous Electrical Activity and Mechanical Hyperalgesia of Myofascial Trigger Spots in Rats. Evid Based Complement Alternat Med.

[85] Chen K, Chung C, Hou T, Kuan S, Chen C, Hong CZ (2001). Inhibitory effect of dry needling on the spontaneous electrical activity recorded from myofascial trigger spots of rabbit skeletal muscle. Am J Phys Med Rehabil.

[86] Koppenhaver SL, Walker MJ, Su J, McGowen JM, Umlauf L, Harrib KD, Ross MD. Changes in lumbar multifidus muscle function and nociceptive sensitivity in low back pain patient responders versus non-responders after dry needling treatment. Man Ther. Available online 13 March 2015 In Press, Corrected Proof 10.1016/j.math.2015.03.003 25801100

[87] Aguilera FJ, Martín D, Masanet R, Botella A, Soler L, Morell F (2009). Immediate effect of ultrasound and ischemic compression techniques for the treatment of trapezius latent myofascial trigger points in healthy subjects: a randomised controlled study. J Manipulative Physiol Ther.

[88] Hendler N, Fink H, Long D (1983). Myofascial syndrome: Response to trigger-point injections. Psychosomatics.

[89] Carlson CR, Okeson JP, Falace DA, Nitz AJ, Lindroth JE (1993). Reduction of pain and EMG activity in the masseter region by trapezius trigger point injection. Pain.

[90] Lim PF, Schmidt J, de Leeuw R, Carlson C, Albuquerque R, Okeson JP (2008). Inability of surface electromyography to register the local twitch response elicited by trigger point injection and snapping palpation in myofascial pain patients. J Musculosk Pain.

[91] Doraisamy MA (2011). Anshul. Effect of Latent Myofascial Trigger Points on Strength Measurements of the Upper Trapezius: A Case-Controlled Trial. Physiother Can.

[92] Celik D, Yeldan I (2011). The relationship between latent trigger point and muscle strength in healthy subjects: a double-blind study. J Back Musculoskelet Rehabil.

[93] Persson AL, Hansson G, Kalliomäki J, Moritz U, Sjölund B (2000). Pressure pain thresholds and electromyographically defined muscular fatigue induced by a muscular endurance test in normal women. Clin J Pain.

